# Beyond Biomarkers: Machine Learning-Driven Multiomics for Personalized Medicine in Gastric Cancer

**DOI:** 10.3390/jpm15050166

**Published:** 2025-04-24

**Authors:** Dongheng Ma, Canfeng Fan, Tomoya Sano, Kyoka Kawabata, Hinano Nishikubo, Daiki Imanishi, Takashi Sakuma, Koji Maruo, Yurie Yamamoto, Tasuku Matsuoka, Masakazu Yashiro

**Affiliations:** 1 Molecular Oncology and Therapeutics, Osaka Metropolitan University Graduate School of Medicine, 1-4-3 Asahimachi, Abeno-ku, Osaka 545-8585, Japanfancanfeng@gmail.com (C.F.); sb24524y@st.omu.ac.jp (T.S.); sn23131y@st.omu.ac.jp (K.K.); sn23089k@st.omu.ac.jp (H.N.); sy23003h@st.omu.ac.jp (D.I.); so22500y@st.omu.ac.jp (T.S.); k.maruo0720@gmail.com (K.M.); yurieyamamoto917@gmail.com (Y.Y.); t22738q@omu.ac.jp (T.M.); 2 Cancer Center for Translational Research, Osaka Metropolitan University Graduate School of Medicine, 1-4-3 Asahimachi, Abeno-ku, Osaka 545-8585, Japan; 3 Department of Gastroenterological Surgery, Osaka Metropolitan University Graduate School of Medicine, 1-4-3 Asahimachi, Abeno-ku, Osaka 545-8585, Japan

**Keywords:** GC, multiomics, personalized medicine

## Abstract

Gastric cancer (GC) remains one of the leading causes of cancer-related mortality worldwide, with most cases diagnosed at advanced stages. Traditional biomarkers provide only partial insights into GC’s heterogeneity. Recent advances in machine learning (ML)-driven multiomics technologies, including genomics, epigenomics, transcriptomics, proteomics, metabolomics, pathomics, and radiomics, have facilitated a deeper understanding of GC by integrating molecular and imaging data. In this review, we summarize the current landscape of ML-based multiomics integration for GC, highlighting its role in precision diagnosis, prognosis prediction, and biomarker discovery for achieving personalized medicine.

## 1. Introduction

Currently, gastric cancer (GC) ranks as the fifth most common malignant cancer and the third leading cause of cancer-related mortality worldwide [1]. While the five-year overall survival (OS) rate of GC patients at stage I achieves a favorable outcome of over 95%, that of GC patients at advanced stages declines sharply [2]. Advanced GC exhibits significant histological and molecular heterogeneity, especially the scirrhous type of GC (SGC), which is histologically characterized by rapid proliferation with extensive stromal fibrosis, showing an extremely poor 5-year OS of 16.2% [3]. On the other hand, the Cancer Genome Atlas (TCGA) project [4] identified four molecular subtypes of GC, including Epstein–Barr virus (EBV), microsatellite instability (MSI), genomically stable (GS), and chromosomal instability (CIN), based on genomic findings. These findings indicated complicated molecular and clinical heterogeneity in GC. Traditional clinical approaches follow a one-size-fits-all paradigm, which is increasingly inadequate in addressing the complex heterogeneity of gastric cancer, underscoring the pressing need for personalized strategies. Current technological innovations in medicine, such as genome-wide sequencing, liquid biopsy, digital histology, and radiology images, are generating vast volumes of individualized data across various omics layers. Effectively harnessing this information requires robust multiomics integration to construct comprehensive molecular and clinical profiles.

ML is a branch of artificial intelligence (AI) that uses algorithms such as support vector machines (SVMs) and random forests to process data, extract features, and generate predictive models, which has undergone rapid technological advancement in recent years [5]. A previous review [6] summarized how ML integrates genomics, epigenomics, transcriptomics, proteomics, and metabolomics, enabling bioinformatics analysis to identify biomarkers and predict the prognosis of GC. Deep learning, a subset of ML using neural network algorithms, can integrate not only clinical data but also medical imaging—such as endoscopy, radiology, and pathology images—to achieve “multidata” to train a predictive model for patient outcomes and prognoses [7]. However, the generalization gap remains a challenge.

**Figure 1:**
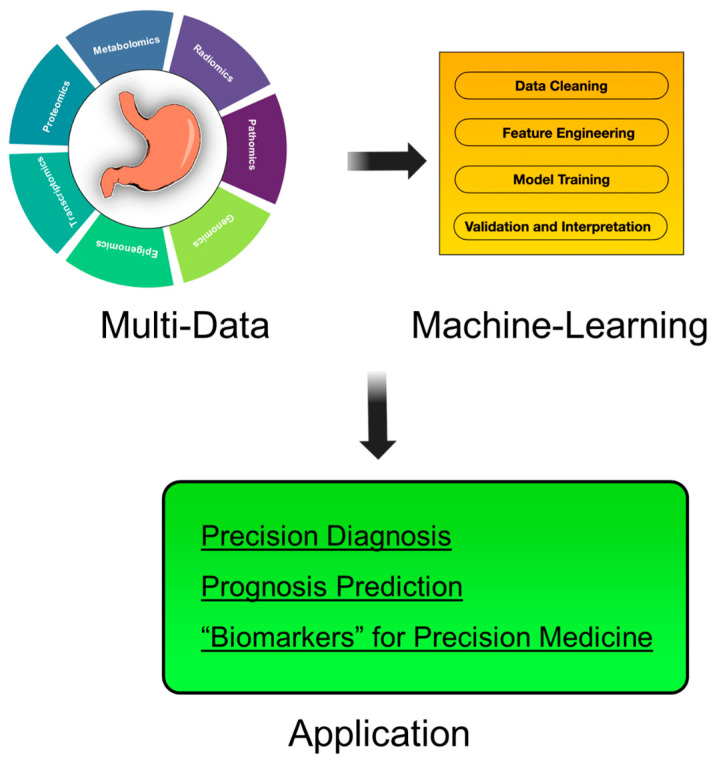
Multiomics and machine learning workflow for personalized medicine: various omics data, including genomics, epigenomics, transcriptomics, proteomics, metabolomics, radiomics, radiomics (endoscopy), and pathomics, are processed through machine learning techniques, leading to applications in precision diagnosis, prognosis prediction, and biomarker discovery.

Here, we focus on recent advancements in the emerging field of ML research in GC, specifically by combining genomics, epigenomics, transcriptomics, proteomics, and metabolomics with different clinical data entities such as pathomics, radiomics (CT), and radiomics (endoscopy). Building upon the cataloging of single-omics applications, our analysis extends to multiomics fusion and practical implementation. These integrated approaches allow us to identify novel biomarkers for personalized medicine, moving beyond traditional biomarkers to incorporate imaging-derived features that significantly enhance predictive capabilities. A key aspect of these advancements is the evolution of computational infrastructure, with GPU acceleration playing a pivotal role in handling the high-dimensional and data-intensive nature of omics-driven computations, particularly in pathomics. However, even with these advances, the hurdles of data harmonization, model interpretability, and clinical translation persist. This review aims to provide a comprehensive summary on leveraging the synergy between multiomics and ML to achieve personalized medicine by advancing precision diagnosis, dynamic prognosis prediction, and biomarker discovery in GC (Figure 1).

## 2. Multiomics Data Types in GC

### 2.1. Imaging-Based Omics

#### 2.1.1. Radiomics (Computed Tomography)

Medical imaging is an indispensable tool in the clinical management of GC, providing crucial insights into tumor staging and disease progression. Computed tomography (CT), as the most widely used and cost-effective imaging modality, plays a pivotal role in noninvasively assessing tumor invasion depth, lymph node (LN) involvement, and distant metastases [8]. Despite its fundamental role in diagnosis and staging, conventional imaging remains largely reliant on qualitative interpretation, with inherent limitations in detecting subtle tumor characteristics. Radiomics has emerged as a transformative approach, bridging this gap by extracting high-dimensional, quantitative imaging features that reflect tumor heterogeneity and biological behavior. By converting routine CT scans into a “virtual biopsy” platform, radiomics enables the identification of subvisual tumor features—such as texture, shape, and intensity variations—that would otherwise be imperceptible to the human eye [9]. Over the past five years, the integration of radiomics with ML has redefined the management of GC through three key evolutionary pathways: (1) detection of occult metastases, (2) profiling of tumor heterogeneity and microenvironment [10], and (3) prognostic modeling and treatment response prediction [11].

Initial efforts focused on detecting LN metastasis, with Dong et al. (2020) [12] analyzing 730 patients across 6 centers in China and Italy, achieving a C-index of 0.797 in external validation for preoperative LN metastasis prediction. Their model outperformed conventional clinical N staging, offering a more precise surgical decision-making basis. Building on this, another study [13] applied deep learning-derived radiomic features to a 959-patient multicenter cohort, refining prognostic stratification for extranodal soft tissue metastases. Furthermore, radiomic signatures [14] were developed by integrating primary tumor and mesenteric fat space features in 177 advanced GC patients, achieving an AUC of 0.835 (testing) for the detection of preoperative occult peritoneal metastases (OPMs). Advancing beyond anatomic staging, Jiang et al. (2023) [15] integrated CT imaging and IHC staining in a tumor microenvironment classifier (N = 2686), achieving AUCs of 0.912 (internal) and 0.909 (external) through in silico validation on independent real-world clinical cohorts, and uncovering significant correlations with chemotherapy and immunotherapy responses (*p* < 0.05), providing a framework for treatment stratification. Prognostication evolved in parallel and a survival CNN model [16] trained on CT images and clinical data from 1061 GC patients was leveraged, achieving a C-index of 0.849. Extending this by integrating a PET radiomics score with clinical staging, the study yielded a C-index of 0.707 [17] in validation for OS prediction. Therapeutic response paradigms closed the loop: Adili et al. (2024) [18] conducted a meta-analysis across 14 studies (N = 3373), demonstrating that combining radiomics and clinical features (C-index = 0.814) significantly enhances neoadjuvant chemotherapy response prediction. Collectively, these studies highlight the transition from conventional imaging-based staging to data-driven, biomarker-enriched models that better capture tumor biology. By integrating radiomics with ML and molecular profiling, these advancements pave the way for more precise risk stratification, tailored treatment strategies, and improved patient outcomes.

#### 2.1.2. Radiomics (Endoscopy)

Endoscopy is an important examination to detect early GC but it remains challenging and often underutilized. The detection rate of early GC (EGC) remains suboptimal, with a miss rate of 20~40% [19]. Conventional grading of endoscopic images is inherently subjective, labor-intensive, and varies widely among endoscopists—particularly less experienced ones. ML, particularly deep learning (DL), has emerged as a transformative tool in medical imaging, with convolutional neural networks (CNNs) demonstrating promising potential in the detection of EGC using endoscopic images [20], improving diagnostic accuracy and reducing interobserver variability.

Recent advancements in convolutional neural network (CNN)-based approaches have shown remarkable promise in endoscopic detection (real-time), classification, and prognosis of gastric neoplasms. A large-scale meta-analysis [21] involving 15 studies demonstrated the feasibility of CNN models across different imaging modalities, with ME-NBI-based systems achieving pooled sensitivities and specificities of 0.95, whereas WLI-based networks showed slightly lower sensitivities (0.80) but comparable specificities (0.95). In a specific task, a recent study [22] employed a CNN-CAD to predict invasion depth, achieving an AUC of 0.94 and surpassing expert endoscopists in accuracy by 17.25%. Moreover, another study [23] applying a CNN-based network achieved 77% accuracy in differentiating intramucosal from submucosal GCs. In pursuit of real-time diagnostic capabilities, a system integrating a YOLO_v3 [24] CNN-based model attained a 95.6% lesion detection rate internally, verified its robustness in both classification and invasion-depth prediction, and demonstrated feasibility for clinical implementation upon external validation. Focusing on high-risk precursors, a BiSeNet [25] architecture was introduced for the real-time segmentation of gastric intestinal metaplasia, yielding an accuracy of 0.96. Beyond isolated image analysis, a multimodal paradigm [26] was constructed by fusing CNN-driven features from static images, image pairs, and videos, demonstrating accuracies of up to 93.55% in diagnosing gastric neoplasms and exemplifying how integrating diverse data sources can bolster clinical decision-making in gastric neoplasm management. These advancements collectively reinforce the pivotal role of ML-driven radiomics (endoscopy) in overcoming the limitations of conventional endoscopy, enhancing early GC detection, and improving patient outcomes through more timely and precise diagnoses.

#### 2.1.3. Pathomics

Pathological examination remains the gold standard for diagnosing GC [27], traditionally relying on labor-intensive histological evaluation to determine tumor morphology, classification, and staging. With the advent of digital pathology, pathologists’ workload has been significantly reduced [28] and large volumes of whole-slide images (WSIs) are now available for computational analysis. Building on these digital resources, deep learning methods have shown considerable promise in extracting morphological signatures from WSIs to assist diagnosis, guide treatment decisions, and even predict critical genomic alterations [29].

A series of deep learning-based approaches has emerged to illustrate a clear progression toward large-scale, multimodal frameworks. Veldhuizen et al. (2023) [30] applied attention-based multiple-instance learning to a TCGA cohort (N = 166), reliably distinguishing intestinal from diffuse GC, with an AUROC of 0.93. Another recent study [31] employed a CNN (Inception-v3) on 639 digital H&E slides from TCGA and Seoul St. Mary’s Hospital, achieving AUCs above 0.87 in identifying MSI-H versus MSS tumors on external validation. Building on these findings, a ResNet-152 architecture was trained on 776 LN slides, detecting metastases with near-perfect AUROCs of up to 0.9994 [32]. These ML models primarily focus on refining diagnostic accuracy, particularly in detecting metastatic GC cells in LNs, significantly alleviating the workload of pathologists. Beyond diagnostic enhancement, deep learning has also demonstrated promising applications in treatment response prediction. For instance, Liu et al. (2024) [33] developed a deep learning ensemble model (ICIsNet) trained on whole-slide images from 264 advanced GC cases, accurately distinguishing responders from nonresponders to first-line PD-1 inhibitor combination chemotherapy (AUC = 0.952), underscoring its potential in personalized treatment planning. Moving toward larger-scale implementations, Wang et al. (2024) [34] introduced CHIEF—trained on over 60,000 whole-slide images—and achieved AUROCs of up to 0.9943 across 15 external test datasets, with a C-index of 0.74 for survival prediction. Finally, a visual–language foundation model, CONCH [34], pretrained on more than 1.17 million histopathology image–caption pairs and biomedical text, displayed state-of-the-art zero-shot performance in classification, segmentation, and cancer subtyping benchmarks. Notably, CONCH’s capacity to integrate histopathological features with textual descriptions hints at a promising shift toward multiomics-driven GC pathology, where combining imaging, transcriptomics, and clinical text data can refine precision diagnostics and biomarker discovery.

### 2.2. Molecular Omics

High-throughput sequencing (HTS), encompassing second- to fourth-generation platforms, has fundamentally transformed multiomics research by enabling massively parallel, scalable, and cost-effective decoding of the genomic and transcriptomic landscapes. Unlike traditional methods such as Sanger or Maxam–Gilbert sequencing, which are limited by low throughput and higher costs, HTS technologies like Illumina and Oxford Nanopore offer the ability to process millions of DNA fragments simultaneously with high sensitivity and specificity, detecting mutations in both coding and noncoding regions, providing the technological foundation for multiomics integration, where sequencing of DNA, RNA, and methylation profiles can be simultaneously leveraged to construct comprehensive molecular models of disease [35].

#### 2.2.1. Genomics

Advances in genome profiling have enabled a more precise dissection of tumor heterogeneity [36]. While identifying actionable alterations has improved targeted therapy in GCs, many patients lack such mutations [37] and resistance remains a persistent challenge. The emergence of single-cell sequencing and ML-driven multiomics integration has provided deeper insights into precise biomarkers for diagnosis, prognosis, and therapy response prediction.

A comprehensive analysis employing NTriPath, support vector machines, and consensus clustering yielded a 32-gene signature (including *TP53*, *BRCA1*, and *MSH6*) that not only demonstrated impressive survival prediction (5-year OS AUC of 0.98) but also stratified both chemotherapy and immunotherapy outcomes, reinforcing its clinical utility. Moreover, the significance of genetic predisposition was underscored through additive logistic regression of a Gansu-based cohort, where 31 SNPs, including rs4823921, were identified and linked to heightened disease incidence and worse outcomes [38]. Meanwhile, multiple supervised and semisupervised learning algorithms (SVM, random forests, KNN, decision tree, neural network, XGBoost) were applied to GEO datasets, leading to the discovery of ESRRG, ATP4A, and ATP4B as powerful diagnostic biomarkers. Notably, the SVM model achieved an AUC of 0.93 on the test set and 0.99 on external validation, underscoring the promise of ML in early detection [39]. Furthermore, an innovative cfDNA-based method called DELFI harnessed genome-wide fragmentation profiles coupled with gradient boosting to detect cancer with high sensitivity (81%) and specificity (98%) [40], offering a minimally invasive screening paradigm. Collectively, these findings illustrate the power of integrating diverse computational pipelines, multigene signatures, and novel molecular assays to enhance early diagnosis, refine prognostic accuracy, and personalize therapeutic strategies in GC.

#### 2.2.2. Epigenomics

Epigenetic reprogramming has emerged as a dynamic regulatory layer in gastric carcinogenesis [41], where ML-driven analysis of DNA methylation patterns, histone modifications, and chromatin accessibility maps is revolutionizing clinical translation. Unlike static genetic alterations, these malleable signatures offer both mechanistic insights into tumor evolution and clinically actionable biomarkers. The growing arsenal of computational approaches—spanning feature selection algorithms (LASSO, Boruta), ensemble classifiers (random forests), and deep learning architectures—now enables systematic mining of epigenetic aberrations in liquid biopsies, premalignant lesions, and tumor microenvironments. This computational–epigenetic synergy is driving three transformative applications: (1) noninvasive early detection through methylation signature deconvolution [42], (2) molecular subtyping linked to stromal reprogramming and therapeutic vulnerabilities, and (3) systemic epigenetic risk stratification [43].

Using LASSO and random forests on plasma cell-free DNA, Qi et al. (2024) [44] identified 21 differentially methylated regions that enabled sensitive and specific early detection (88.38% and 94.23%, respectively) of GC, thereby offering a robust liquid biopsy solution for clinical screening. Another recent study [45] employed Boruta feature selection and random forests on cfDNA methylation data, characterizing 67,832 differentially methylated regions across gastrointestinal malignancies, and culminating in the EpiPanGI Dx panel, with a multicancer prediction accuracy of 0.85–0.95. In contrast, significant aberrant methylation events in nodular gastritis were identified using BeadChip arrays and RNA-Seq; hierarchical clustering of 585 methylation-resistant promoter CpG islands revealed a distinct sample cluster with strong methylation induction, suggesting an early pathogenic mechanism [46]. Furthermore, by integrating non-negative matrix factorization, LASSO Cox, SVM, and ANN on TCGA and GEO datasets, Wu et al. (2023) [47] uncovered key tumor-microenvironment- and epigenetics-associated genes (e.g., SRMS, MET) that refine prognostic stratification and may shape immunotherapy responses. Finally, leveraging nearest template prediction alongside ATAC-Seq, ChIP-Seq, and single-cell RNA-Seq, Ho et al. (2023) [48] classified TCGA and ACRG GC samples into mesenchymal-type molecular subtypes and identified TEAD1- and NUAK1-driven enhancers, underscoring novel therapeutic opportunities in aggressive tumor subgroups. Collectively, these findings underscore the vital role of epigenetic mechanisms in advancing early detection, refining prognosis, and guiding targeted interventions for GC.

#### 2.2.3. Transcriptomics

Transcriptomic profiling has become a cornerstone of personalized medicine in GC, with ML revolutionizing its analytical potential. Modern ML algorithms excel at decoding high-dimensional RNA data—spanning protein-coding genes, noncoding RNAs (ncRNAs), and immune microenvironment signatures—to uncover clinically actionable patterns [49]. By integrating bulk and single-cell RNA sequencing with multimodal datasets (e.g., genomic, proteomic, clinical), ML models systematically identify diagnostic biomarkers, prognostic signatures, and immune correlates while addressing transcriptomic heterogeneity [50]. These approaches not only pinpoint dysregulated genes and ncRNAs but also decode their interplay with tumor progression, therapy resistance, and immune evasion.

Using multiple classifiers (SVM, decision trees, MLP, XGBoost) on GEO datasets, one study identified COL1A1 and LUM as hub diagnostic genes, with XGBoost achieving an AUC of 0.9922 in the training set and MLP yielding 0.9082 in the test set [51]. Another recent study applied LASSO and SVM-RFE to combined GEO, TCGA, and GTEx data, unveiling ABCA8, COL4A1, FAP, LY6E, MAMDC2, and TMEM100 as robust diagnostic markers (AUC > 0.707) [52]. In contrast, a random forest-based NanoString transcriptomics approach was constructed to derive a 19-gene signature predictive of paclitaxel’s survival advantage, achieving an external validation AUC of 0.88 and underscoring the value of tailored therapies [53]. Moreover, the integration of LASSO, SVM, random forest, and XGBoost analyses on serum and tumor tissue identified miR-1290, miR-1246, and miR-451a as significantly upregulated miRNAs in GC, while miR-187 emerged as an independent prognostic factor, reinforcing their clinical utility in patient survival assessment [54]. Furthermore, through information gain and logistic regression, downregulated LOC441461 was found to be correlated with advanced TNM stage and poorer survival, emphasizing its relevance for risk stratification [55]. From a mechanistic perspective, multiplex immunohistochemistry integrated with machine learning algorithms revealed PCDHGA10 as an independent prognostic factor associated with key immune cells (e.g., CD8+ T cells, Foxp3+ Tregs, CD68+ macrophages) and immune checkpoints (e.g., CTLA-4, LAG-3, PD-L1), suggesting a broader immunomodulatory function [56]. Collectively, these findings illuminate how ML-driven transcriptomics approaches can uncover both diagnostic and prognostic biomarkers, deepening our understanding of the tumor immune microenvironment and advancing personalized strategies in GC management.

#### 2.2.4. Proteomics

Proteomics technologies, synergized with ML algorithms, are redefining biomarker discovery and clinical translation in GC research [57]. The integration of high-throughput platforms such as LC-MS/MS, TMT labeling, and Olink Proteomics with computational models—including LASSO, XGBoost, and SVM—has enabled the systematic identification of stage-specific protein signatures linked to tumor initiation, progression, and metastasis. By correlating dynamic proteome profiles with clinical outcomes, researchers can now stratify patients based on molecular subtypes, predict their treatment responses, and uncover actionable targets.

Using LC-MS/MS with TMT labeling, Zhou et al. (2020) [58] identified FR2, PCSK9, MGP, and SOD1 as differentially expressed proteins in early-stage disease, constructing a logistic regression model that initially achieved an AUC of 1.00 but yielded a more realistic AUC of 0.711 upon leave-one-out cross-validation. Another recent study [59] exploited random forests and LASSO regression on plasma samples to uncover GSTP1, CSRP1, and LY6G6F, underscoring the clinical utility of proteomic signatures in distinguishing cardia GC from precancerous lesions. Moreover, Li et al. (2024) [60] used VSOLassoBag and XGBoost to pinpoint five key proteins (CDHR2, ICAM4, PTPRM, CDC27, and FLT1) that effectively differentiated healthy individuals, precancerous lesions, and cardia GC (AUC = 0.931), with functional pathways implicating these markers in cell adhesion, growth regulation, and angiogenesis. In contrast, another study [61] harnessed SVM and WGCNA on TCGA and Plasma Proteome Database data to identify CST1 and INHBA as significantly upregulated genes in stomach cancer, and their SVM classifier (AUC = 0.9924) highlighted the robust diagnostic and prognostic potential of these genes. Meanwhile, a CBP-related prognostic signature was tested using LASSO on TCGA and ACRG datasets—incorporating LOX, CP, and other copper proteome components—that were correlated with poorer survival, suggesting a mechanistic link to cuproptosis pathways, and with an AUC of 0.75 for predicting five-year overall survival [62]. Building on this prognostic focus, XGBoost was applied to reveal a 10-protein signature (including DUOXA2, ITGA7, and TADA1) predictive of peritoneal metastasis [63], while tumor-infiltrating immune cell analysis via CIBERSORT underscored potential crosstalk between these proteins and the immune microenvironment. Extending to therapy response, Sun et al. (2024) [57] leveraged high-resolution mass spectrometry and SVM modeling to identify 10 biomarkers—such as COL15A1, SAMHD1, and VWF—that accurately predicted PD-1 inhibitor response (AUC = 0.97), thereby fostering precision immunotherapy for advanced cases. Finally, Olink Proteomics was utilized to show that MUC16 was significantly elevated in metastatic advanced GC, with random forest (AUC = 0.76) and decision tree algorithms [64] as well as ELISA validation, confirming its prognostic relevance for high-risk patients. Together, these findings illustrate how sophisticated proteomics strategies and ML can elucidate disease mechanisms, enhance risk stratification, and ultimately pave the way for more effective, individualized interventions in GC.

#### 2.2.5. Metabolomics

Metabolomics, positioned as the functional endpoint of omics cascades, provides a dynamic lens to decode the metabolic rewiring that fuels gastric carcinogenesis, progression, and therapeutic resistance [65]. Unlike upstream molecular layers, metabolomics captures real-time biochemical activity, directly reflecting the interplay of genetic, environmental, and therapeutic perturbations. In GC, dysregulated pathways—spanning energy metabolism, redox homeostasis, and nucleotide/lipid biosynthesis—serve as both biomarkers and actionable targets. Recent technological leaps in high-resolution mass spectrometry and ML-driven data integration have transformed metabolomics into a cornerstone of personalized medicine, enabling the discovery of diagnostic, prognostic, and predictive signatures [66].

By integrating untargeted and targeted metabolomics in a large Korean cohort, Han et al. (2022) [67] discovered significantly lower levels of L-carnitine and citric acid in individuals who ultimately developed GC, suggesting compromised energy metabolism as an early hallmark of malignant progression. Another recent study [68] leveraged targeted lipidomics with an XGBoost framework, identifying 11 plasma lipid signatures (e.g., PC38:6) that markedly improved diagnostic accuracy (AUC = 0.97), thereby reinforcing the power of lipidomics-based screening. Focusing on subtype-specific detection, Wei et al. (2024) [69] employed OPLS-DA and Cox regression to construct a 25-metabolite panel (AUC up to 0.90) for gastric cardia adenocarcinoma, highlighting the nuance of metabolome-driven precision diagnosis. Moreover, Chen et al. (2024) [70] used LASSO and random survival forest approaches on a multicenter dataset to develop both a 10-metabolite diagnostic model (AUC = 0.967) and a 28-metabolite prognostic model (C-index = 0.90), emphasizing the dual utility of metabolomics data in detection and outcome prediction. In contrast, Kaji et al. (2020) [71] identified reduced β-alanine as a marker of elevated peritoneal recurrence risk, pointing to potential disruptions in protective metabolic pathways and unveiling a new target for postoperative surveillance. Similarly, hierarchical clustering of metabolites such as lysophosphatidic acid and sphingosine-1-phosphate was employed to forecast postoperative recurrence, offering a refined risk stratification tool [72]. Moving to therapeutic mechanisms, mass spectrometry-based pseudotargeted metabolomics in MGC-803 cells was used to reveal shifts in aspartate, tryptophan, succinate, uridine, and cytidine under chemotherapeutic stress, providing mechanistic insights into adaptive metabolic reprogramming [73]. For targeted therapies, Wang et al. (2024) [74] applied k-means clustering and Simpson’s diversity index in the VARIANZ study to identify subpopulations sensitive to trastuzumab. Lastly, by applying random forest modeling to serum profiles, a predictive model was constructed for malnutrition risk (AUC = 0.702), underscoring the broader clinical significance of metabolomics in monitoring patients’ nutritional status [75]. Collectively, these findings reflect the multifaceted potential of metabolomic profiling across the continuum of GC management, from pinpointing early biomarkers and refining prognostic assessments to informing targeted treatment and holistic patient care. Table 1 summarizes the applications of genomics, epigenomics, transcriptomics, proteomics, metabolomics, pathomics, radiomics, and radiomics (endoscopy) in ML for GC.

## 3. ML for GC Research

### 3.1. Multiomics Integration

As single-omics approaches can be limited by incomplete perspectives, **multiomics integration** has gained traction to provide a more comprehensive understanding of GC. There are three primary strategies for integrating multiomics data [82]: **cascade-based integration (early integration)**, where omics data matrices are combined into a single large dataset before analysis; **transformation-based integration (intermediate integration)**, where each omics dataset is transformed into a structured format such as a graph or core matrix before modeling; and **model-based integration (late integration)**, where separate models are trained on different datasets and then merged into a final predictive model. These methodological advancements have catalyzed a paradigm shift in GC research, enabling systematic dissection of molecular mechanisms and clinically actionable biomarker discovery.

Capitalizing on these integrative strategies, recent multiomics investigations have substantially advanced our understanding of GC’s pathogenesis and prognosis. Foundational studies first demonstrated the power of cross-omics synergy: Zhang et al. (2022) [83] combined DNA and RNA sequencing to delineate early metastatic drivers such as MADCAM1 and TP53, while another study [84] identified *GSDMC* overexpression via genomic and transcriptomic analyses of the 8q24.21 region, linking this alteration to aggressive phenotypes. Building on these discoveries, Liu et al. (2024) [85] integrated transcriptomic (including H. pylori-associated RNA-seq [86] and TCGA-STAD), genomic (SNV, CNV, methylation), and single-cell datasets (GSE134520) to comprehensively investigate the ferroptosis-related gene *YWHAE* and its association with cancer-critical pathways (MAPK, NF-κB, and PI3K), immune microenvironment modulation, and prognostic modeling. Their prognostic risk score was constructed and then validated across multiple cohorts, yielding AUC values between 0.58 and 0.81. Furthermore, another study [87] focused on cancer-associated fibroblasts, establishing a robust prognostic model that incorporated three hub genes—including *CDH6*, a gene highly expressed in nonresponsive patients—and demonstrated survival prediction accuracies exceeding 0.75 for the 1-, 3-, and 5-year endpoints. From an immune profiling perspective, a high level of *PCDHGA10* expression was identified using genomic analysis and multiplex immunohistochemistry, correlating with increased infiltration of CD8+ T cells, CD68+ macrophages, Foxp3+ T cells, and CD4+ T cells, and achieving an AUC of 0.838 for prognosis prediction [56]. Collectively, these investigations underscore how multiomics fusion—encompassing genomics, epigenomics, transcriptomics, and proteomics, as well as emerging fields such as pathomics—enables precise molecular stratification, robust predictive modeling, and actionable therapeutic insights in GC.

### 3.2. ML Algorithms

GC research increasingly integrates multiomics data, including radiomics (CT, MRI, and endoscopy), pathomics, genomics, epigenomics, transcriptomics, proteomics, and metabolomics. ML methods facilitate the extraction of clinically relevant insights from these complex datasets and can be broadly categorized into four types [88]: supervised learning, unsupervised learning, reinforcement learning, and deep learning. Supervised learning, including support vector machines (SVMs), random forests (RFs), decision trees, and k-nearest neighbors (KNN), is widely used for classification and regression tasks based on labeled data. Unsupervised learning, such as k-means clustering, hierarchical clustering, principal component analysis (PCA), t-distributed stochastic neighbor embedding (t-SNE), and autoencoders, uncovers hidden patterns in unlabeled data. Meanwhile, deep learning architectures such as convolutional neural networks (CNNs), recurrent neural networks (RNNs), transformers, and graph neural networks (GNNs) are particularly effective in analyzing high-dimensional omics data [89] for prognosis or therapeutic targets.

### 3.3. Computational Hardware Requirements (GPU)

The rapid evolution of ML has been closely tied to advances in hardware performance, where ultrahigh computational power and real-time processing capacity have made large-scale data parallelization feasible [90]. Pathomics has underscored the importance of robust GPU resources, as whole-slide images (WSIs) typically exceed one gigabyte in size and can contain over a billion pixels. These massive datasets necessitate substantial GPU memory, rendering high-end graphics cards indispensable for local ML development in medical research centers. Many research centers mitigate memory constraints by segmenting large whole-slide images (WSIs) into smaller patches; in contrast, facilities with extensive GPU clusters can process an entire WSI in a single pass. Alongside high-performance computing (HPC) systems, institutions may alternatively turn to cloud-based GPU rentals, which offer flexibility and lower short-term costs but raise concerns regarding data privacy and research ethics when patient data might be uploaded [91]. Table 2 provides an overview of current GPU configurations and performance metrics across different research centers involved in pathomics projects.

## 4. ML-Driven Multiomics for Personalized Medicine in GC

### 4.1. Precision Diagnosis

The integration of ML into GC diagnostics has evolved into a multifaceted framework that spans the entire diagnostic continuum. Beyond enhancing early detection, ML now orchestrates four synergistic technological pillars: endoscopic image analysis, liquid biopsy biomarker discovery, advanced imaging interpretation, and computational pathology optimization. This paradigm shift addresses critical gaps across lesion identification, molecular subtyping, metastasis detection, and treatment response prediction [99].

#### 4.1.1. Endoscopy-Driven Diagnosis

For instance, convolutional neural networks (CNNs) can identify subtle mucosal irregularities [23] and predict tumor invasion depth with AUCs exceeding 90% [22]. These models are particularly valuable for detecting early-stage GC.

#### 4.1.2. Liquid Biopsy and Multiomics Biomarkers

Beyond imaging, ML integrates transcriptomics, epigenomics, proteomics, and metabolomics data from blood samples to identify noninvasive biomarkers for early detection. For example, targeted metabolomics identified 11 plasma lipid signatures with an AUC of 0.97 for GC detection, offering a noninvasive diagnostic approach [68].

#### 4.1.3. Pathomics for Definitive Diagnosis

Histopathology remains the gold standard for cancer diagnosis, and pathomics models are now used to automate the quantification of nuclear morphology, glandular architecture, and stromal interactions. In previous research, the CHIEF foundation model demonstrated robust cancer classification (AUROC = 0.939) and genetic mutation prediction (AUROC > 0.8 for key oncogenic mutations) [34].

### 4.2. Prognosis Prediction

Contemporary prognostic stratification in GC has transcended conventional clinicopathological paradigms, ushering in an era of personalized medicine frameworks powered by multimodal ML architectures. The integration of longitudinal multiomics data streams now enables real-time prognostic refinement through three transformative dimensions [100]:

#### 4.2.1. Molecular Biomarkers

One notable application involves **cell-free multiomics profiling**, where genomics, epigenomics, and transcriptomics data from circulating cfDNA and cfRNA have been utilized to establish a **plasma-based prognostic model**. This model demonstrated superior sensitivity in detecting tumor-associated alterations, with cfRNA-derived signatures outperforming cfDNA in predicting survival outcomes [101].

#### 4.2.2. Treatment Response Prediction

A recent study systematically integrated transcriptomics, proteomics, and metabolomics to classify patients into quiescent, glycolysis/gluconeogenesis (GG), alanine–aspartate–glutamate (AAG), and mixed metabolic subtypes. The GG subtype exhibited the worst prognosis but the highest sensitivity to chemotherapy, whereas the quiescent and AAG subtypes demonstrated enhanced response to immune checkpoint inhibitors [102].

### 4.3. “Biomarkers” for Personalized Medicine

Biomarkers have long been central to guiding diagnosis, prognosis, and therapeutic choices in GC [103]. However, the inherent heterogeneity of this disease often renders single-molecule approaches insufficient. Recent advancements in ML and imaging technologies are driving a paradigm shift toward integrating imaging-derived features from digital pathology and radiology [104]. These imaging-based features capture tumor heterogeneity and phenotypic characteristics beyond what molecular markers alone can achieve, enabling more precise risk stratification and treatment optimization.

#### 4.3.1. Advancements in Imaging-Based “Biomarkers”

ML identifies nontraditional biomarkers such as imaging-derived features. A recent study developed a multiscale attention-based network trained on hematoxylin and eosin (H&E)-stained whole-slide images, which generated a digital pathology signature (DPS) predictive of GC recurrence [105].

#### 4.3.2. Imaging for Characterization

CT remains the cornerstone for assessing locally advanced tumors and distant metastases. A radiomics nomogram integrating CT-derived texture features achieved AUCs exceeding 0.8 in predicting peritoneal metastasis preoperatively, enhancing diagnostic accuracy [106]. CT-based deep learning models can noninvasively infer molecular subtypes, such as microsatellite instability (MSI) status in GC. A radiomics–clinical combined model integrating clinical and quantitative CT features achieved AUCs of over 0.75 [107].

## 5. Challenges and Limitations

The integration of ML and multiomics data in GC research holds immense promise but remains fraught with obstacles that limit its routine clinical application. These challenges range from fundamental technical bottlenecks—such as data heterogeneity and limited sample sizes—to broader clinical and ethical issues, including model interpretability, regulatory constraints, and biases embedded within algorithms [108].

### 5.1. Technical Challenges

A fundamental challenge in integrating multiomics data for ML applications is the inherent heterogeneity of these datasets [109]. Differences in data dimensionality—including imaging, molecular, and clinical omics, along with variations in sampling frequency and quality—create significant barriers to seamless integration. Moreover, radiomics features extracted from CT and MRI scans often exhibit inconsistencies when compared to genomics and transcriptomics data, necessitating rigorous normalization and alignment to mitigate batch effects across sequencing platforms. Compounding this complexity, small sample sizes remain a critical limitation, as seen in cohorts such as TCGA-STAD, where the number of cases is insufficient for training high-capacity deep learning models. While techniques such as synthetic data augmentation and transfer learning have been employed to circumvent these constraints, they risk introducing biases that compromise model reliability. Furthermore, model generalizability continues to be a concern, as algorithms trained on homogeneous datasets—often derived from single institutions with specific population characteristics—fail to replicate their performance across diverse cohorts with distinct molecular landscapes. Meanwhile, federated learning frameworks, inconsistencies in data governance, and the computational overheads associated with secure multi-institutional collaborations present persistent obstacles [110].

### 5.2. Clinical Translation Barriers

Beyond the laboratory, the clinical translation of ML-based multiomics tools in GC remains fraught with challenges [111]. A primary concern is the inherent lack of interpretability in deep learning models, often perceived as “black-box” systems, which undermines clinicians’ trust and limits their integration into routine practice [112]. Furthermore, regulatory and validation hurdles persist, as ML-driven diagnostic and prognostic tools must undergo rigorous, prospective clinical trials within the stringent frameworks of regulatory agencies such as the FDA and CE. These barriers have thus far precluded the approval of any ML-based system specifically designed for GC, underscoring the need for standardized validation protocols and robust real-world evidence.

### 5.3. Ethical and Regulatory Issues

Efforts to harness ML and multiomics tools at scale often encounter ethical and regulatory challenges, particularly concerning patient privacy, algorithmic bias, and commercialization [113]. One primary concern is the potential for ML models to inherit and exacerbate biases present in training data [114]. For example, models predominantly trained on East Asian cohorts may exhibit diminished predictive accuracy when applied to African or European populations, thereby reinforcing existing health disparities [115]. Furthermore, ensuring patient privacy while utilizing extensive clinical datasets remains a critical issue. Even with deidentification methods, residual risks persist due to variations in institutional data structures and the potential for reidentification through cross-referenced datasets. Therefore, the ethical deployment of ML in multiomics research necessitates rigorous bias mitigation strategies, transparent model validation across diverse populations, and robust regulatory frameworks to safeguard patient confidentiality.

## 6. Future Directions

Looking ahead, efforts should focus on enhancing the interpretability and generalizability of ML-driven multiomics models for GC, particularly through the adoption of explainable AI frameworks and robust multi-institutional collaborations. Standardized data collection and harmonization protocols—spanning genomics, proteomics, imaging, and clinical metadata—are critical to mitigate batch effects and reduce biases rooted in population heterogeneity [116]. Ultimately, the synergy of next-generation ML algorithms, coupled with interdisciplinary partnerships among clinicians, data scientists, and regulatory bodies, holds promise for a seamless translation of multiomics-driven personalized medicine into routine GC care.

## 7. Conclusions

This review highlights the growing promise of ML in transforming GC care through the integration of imaging-, molecular-, and clinical-based multiomics data. ML-driven multiomics has demonstrated potential in precision diagnosis, prognosis prediction, and biomarker discovery. Nevertheless, the field faces obstacles related to data heterogeneity, model interpretability, and ethical and regulatory constraints. With continued advancements in data integration, validation, and interdisciplinary collaboration, ML-driven multiomics is set to become an indispensable force in shaping the future of personalized medicine for GC.

## Figures and Tables

**Table 1:** Summary of studies in machine learning with multiomics.

Omics	Author (Year)	Data Source	Sample Type and Size	Method	Task	Biomarkers	Performance Metrics
Radiomics	Jiang et al. (2023) [15]	Multicenter study (Southern Medical University, Stanford University, Sun Yat-sen University Cancer Center, Guangdong Provincial Hospital of Chinese Medicine)	CT imaging (GC patients, N = 2686)	Deep learning (CNN-based)	Treatment response prediction	Deep learning-based image features	AUC = 0.722
Radiomics	Tao et al. (2024) [76]	West China Hospital, Sichuan University, The First Affiliated Hospital of Chengdu Medical College, People’s Hospital of Leshan	CT imaging (GC patients, N = 771)	Deep learning (vision transformer-based)	Diagnosis prediction (T stage: T1–T2 vs. T3–T4)	Deep learning features (1280 features) combined with radiomics features (512 features)	AUC = 0.972
Radiomics (Endoscopic)	Zhu et al. (2019) [22]	Endoscopy Center, Zhongshan Hospital, Fudan University	Endoscopic images (N = 790)	Deep learning (CNN-based)	Diagnosis prediction (invasion depth)	Deep learning-based image features	AUC = 0.94
Radiomics (Endoscopic)	Liu et al. (2022) [77]	Shanghai General Hospital-South and Shanghai Jiao Tong University Affiliated Sixth People Hospital	Endoscopic images (N = 6177)	Deep learning (CNN-based)	Diagnosis prediction (gastric neoplastic lesions)	Deep learning-based image features	AUC = 0.928
Pathomics	Veldhuizen et al. (2023) [30]	TCGA	WSIs (N = 166)	Deep learning	Diagnosis prediction (diffuse vs. intestinal)	Deep learning-based histopathology features	AUROC: 0.93
Pathomics	Saldanha et al. (2023) [78]	Four patient cohorts from Switzerland, Germany, the UK, and the USA	WSIs (N = 60,530)	swarm learning	Diagnosis prediction (MSI, EBV status)	Deep learning-based histopathology features	AUROC: 0.8092, 0.8372 (MSI, EBV prediction, respectively)
Genomics	Cheong et al. (2022) [79]	TCGA, GEO, ACRG, Yonsei cohort	Tissue (N = 567)	NTriPath, SVM	Prognosis prediction	32-gene signature (including TP53, BRCA1, MSH6, PARP1, ACTA2)	AUC = 0.981
Genomics	Wu et al. (2023) [47]	TCGA-STAD, GEO (GSE84437, GSE54129, GSE65801)	Tissue (N = 443)	NMF, SVM, neural networks, LASSO	Prognosis prediction (OS)	SRMS, MET, OLFML2B, KIF24, CLDN9, RNF43, NETO2, PRSS21	AUC > 0.7
Epigenomics	Kandimalla et al. (2021) [45]	TCGA, GSE72872	Plasma (N = 300)	Random forest	Diagnosis prediction	3 DMR panels	AUC = 0.90
Epigenomics	Li et al. (2020) [80]	GEO, TCGA	Tissue (N = 368)	LASSO	Prognosis prediction (OS)	TREM2, RAI14, NRP1, YAP1, MATN3, PCSK5, INHBA, MICAL2	AUC = 0.74
Transcriptomics	Kong et al. (2022) [81]	TCGA, STRING database	Tissue (N ≥ 700)	Network-based machine learning	Treatment response prediction (ICI)	Network-derived transcriptomic biomarkers	AUC = 0.72
Transcriptomics	Lee et al. (2022) [55]	TCGA-STAD, UCSC Xena	Tissue (N = 379)	Hierarchical clustering	Prognosis prediction	LOC441461	other
Proteomics	Li et al. (2024) [60]	Multicenter study (China)	Serum (N = 60)	XGBoost	Diagnosis prediction (CGC vs. healthy control)	CDHR2, ICAM4, PTPRM, CDC27, FLT1	AUC = 0.931
Proteomics	Sun et al. (2024) [57]	First Affiliated Hospital of Zhengzhou University	Tissue (N = 28)	SVM, Boruta	Treatment response prediction (ICIs)	COL15A1, SAMHD1, DHX15, PTDSS1, CFI, ORM2, VWF, APOA1, EMC2, COL6A2	AUC = 0.96
Metabolomics	Liu et al. (2022) [68]	National Upper Gastrointestinal Cancer Early Detection Program (China)	Plasma (N = 200)	OPLS-DA	Diagnosis or prognosis prediction	PC38:6(20:4), PC38:5(20:4), PC34:3, LysoPC18:3, LysoPC20:4, LPI18:0, LPI20:4, FFA20:4 (arachidonic acid), FFA18:3 (α-linolenic acid), FFA18:0 (stearic acid), PA32:1	AUC = 0.97(for diagnosis) 0.82(for prognosis)
Metabolomics	Chen et al. (2024) [70]	Multicenter plasma metabolomics dataset (China)	Plasma (N = 702)	LASSO, random forest, SVM	Diagnosis prediction (GC vs. NGC)	Succinate, Uridine, Lactate, SAM, Pyroglutamate, 2-Aminooctanoate, Neopterin, GlcNAc6p, Serotonin, NMN	AUC = 0.967

EBV (Epstein–Barr Virus), TCGA (The Cancer Genome Atlas), ACRG (Asian Cancer Research Group), GEO (Gene Expression Omnibus), NMF (non-negative matrix factorization), SVM (support vector machine), LASSO (least absolute shrinkage and selection operator), DMR panels (differentially methylated region panels), XGBoost (extreme gradient boosting), ICIs (immune checkpoint inhibitors), NGC (nongastric cancer), CGC (cardia gastric cancer).

**Table 2:** Summary of GPUs used in deep learning of pathomics.

Author (Year)	University, Country	Task	Dataset Size	Model	Patch Size	Batch Size	GPU Type (Memory)	Training Epochs	Performance Metrics
Lu et al. (2024) [92]	Harvard Medical School, USA	Zero-shot visual-language pathology AI	21,442 WSIs	CONCH	448 × 448 px	1536 patches	8 × NVIDIA A100 (80 GB each)	40 epochs	Zero-shot accuracy: 91.3%
Wang et al. (2024) [34]	Harvard Medical School, USA	Cancer diagnosis and prognosis prediction	60,530 WSIs	CHIEF	256 × 256 px	1 WSI	8 × NVIDIA V100 (32 GB each)	50 epochs	C-index: 0.74
White et al. (2024) [93]	Mater Misericordiae University Hospital, Ireland	Biopsy prioritization	24,983 WSIs	MIL	512 × 512 px	Not specified	8 × NVIDIA V100 (32 GB)	200 epochs	F1 Score: 0.949
Gustav et al. (2024) [94]	Technical University Dresden, Germany	Predicting MSI and POLE mutations in colorectal cancer	2039 WSIs	Vision Transformer	Not specified	Not specified	NVIDIA RTX A6000 (48 GB)	Not specified	AUROC: 0.94
Hilgers et al. (2024) [95]	Technical University Dresden, Germany	Automated curation of WSIs	32,975 WSIs	ResNet18	224 × 224 px	128 patches	NVIDIA RTX A6000 (48 GB)	500 epochs	AUROC: 0.995
Liu et al. (2024) [94]	Sun Yat-sen University, China	Predicting response to PD-1 blockade in advanced GC	313 WSIs	DenseNet121, EfficientNet-B4, Swin V2	1024 × 1024 px	32 patches	2 × NVIDIA RTX 3090 (24 GB each)	100 epochs	AUROC: 0.92–1.00
Yang et al. (2024) [96]	Wenzhou Medical University, China	Prognosis and treatment response prediction	1481 WSIs	OCDPI	224 × 224 px	8 patches	NVIDIA RTX 4090 (24 GB)	40 epochs	Not specified
Huang et al. (2024) [97]	Southeast University, China	Morphological profiling of CRC organoids	31,360 bright field images + 17,000 fluorescent images	Generative	1360 × 1024 px	Not specified	NVIDIA RTX 3090 (24 GB)	200 epochs	Not specified
Choudhury et al. (2024) [98]	University of Chicago, USA	HPV status prediction	941 WSIs	Xception-based CNN	299 × 299 px	Not specified	NVIDIA Titan RTX (24 GB)	1 epoch	AUROC: 0.84

WSIs (whole slide images), MIL (multiple instance learning), CRC (colorectal cancer), HPV (human papillomavirus).

## Data Availability

No new data were created in this study. Data sharing is not applicable to this article.
